# Beneficial Effects of Epigallocatechin Gallate in Preventing Skin Photoaging: A Review

**DOI:** 10.3390/molecules29225226

**Published:** 2024-11-05

**Authors:** Jiaqiang Sun, Yuelu Jiang, Jing Fu, Linlin He, Xinmiao Guo, Hua Ye, Cuiyuan Yin, Hongbo Li, Heyuan Jiang

**Affiliations:** 1 College of Biological Science and Engineering, Shaanxi University of Technology, Hanzhong 723001, China; sunjiaqiang24@163.com (J.S.); fujing@snut.edu.cn (J.F.); 18717215515@163.com (X.G.); yh18716289047@163.com (H.Y.); 15691611707@163.com (C.Y.); 2 Shaanxi Province Key Laboratory of Bio-Resources, Shaanxi University of Technology, Hanzhong 723001, China; 3 Qinba Mountain Area Collaborative Innovation Center of Bioresources Comprehensive Development, State Key Laboratory of Biological Resources and Ecological Environment (Incubation), Hanzhong 723001, China; 4 College of Food Science and Engineering, Tianjin University of Science and Technology, Tianjin 300457, China; 18969036236@163.com (Y.J.); hljbobo@tust.edu.cn (H.L.); 5 Key Laboratory of Special Economic Animal and Plant Biology and Genetic Breeding, Ministry of Agriculture and Rural Affairs, Tea Research Institute, Chinese Academy of Agricultural Sciences, Hangzhou 310008, China

**Keywords:** ultraviolet (UV), skin photoaging, epigallocatechin gallate (EGCG), beneficial effects

## Abstract

Skin photoaging, primarily caused by ultraviolet (UV) radiation, leads to skin metabolic disorders, which have adverse psychological and physiological effects on individuals. However, traditional medications for repairing skin photoaging cause side effects. Natural bioactive compounds have been shown to prevent and treat skin photoaging with fewer side effects. Epigallocatechin gallate (EGCG), the main substance in tea polyphenols, is a natural bioactive compound with a range of properties. This review summarizes the beneficial effects and mechanisms of EGCG, as well as the application forms of EGCG in repairing photoaged skin. Results indicated that EGCG has repair effects, including improving elasticity, enhancing moisturization, inhibiting damage, and reducing pigmentation of photoaged skin. It has also been demonstrated that EGCG delivery systems, modified EGCG, and combinations with other bioactive substances could be used for repairing photoaged skin due to its poor stability and low bioavailability. EGCG effectively repairs various types of skin damage caused by UV radiation while maintaining normal skin structure and function. It is, therefore, an effective candidate for repairing photoaged skin. These results could provide references for the development and application of EGCG products for the treatment of photoaged skin.

## 1. Introduction

Skin photoaging refers to the premature aging of human skin resulting from exposure to ultraviolet (UV), artificial, visible, and infrared lights, with UV radiation being the most important factor [1,2]. The skin plays a beneficial role in absorbing UV radiation for the body, facilitating vitamin D synthesis, and maintaining homeostasis of the body [3]. However, the adverse effects of prolonged UV exposure on the skin are well documented. The UV radiation that reaches Earth’s surface primarily consists of UVB (290~320 nm) and UVA (320~400 nm) rays, among these, UVA rays penetrate more deeply into the dermis [1,4]. Most UVB is absorbed by the stratum corneum of the epidermis, while a small fraction passes through this layer to affect the upper portion of the dermis [1]. The mechanisms underlying skin photoaging induced by these two different wavelengths may vary. Initially, UVB is absorbed by chromophores in skin cells, leading to DNA photolesions and generating small amounts of reactive oxygen species (ROS). In contrast, UVA predominantly induces oxidative damage by enhancing ROS production, which results in lipid oxidation and subsequently causes indirect DNA damage [5]. Human skin is simultaneously exposed to both wavelengths of UV radiation, which collectively contribute to photoaging processes. Ultimately, these factors lead to loose, less elastic skin that appears dry and flaky with localized pigmentation changes and may even provoke malignant proliferation of skin cells [6]. The probability of human skin photoaging has increased due to ozone layer depletion, a pressing health concern for humanity. Unlike other diseases, skin photoaging imposes considerable social pressure along with a range of physical and psychological effects.

Topical retinoids (tretinoin, tazarotene, adapalene, etc.) effectively reduce the signs of skin aging caused by UV radiation, such as wrinkles, reduced elasticity, and hyperpigmentation [4,7]. High concentrations of α-hydroxy acids are often used in chemical peels to improve photodamaged skin by exfoliating the epidermal stratum corneum and inducing collagen synthesis [8,9]. However, these medications have significant side effects such as skin irritation, redness, peeling, and in severe cases, scarring. Therefore, there remains a demand for the prevention and repair of photoaged skin, which stimulates the search for safe, reliable, and effective drugs [10].

Natural bioactive substances have emerged as one of the main candidates due to fewer side effects and diverse biological activities compared with commonly used medicines for treating photoaged skin. Studies have demonstrated that natural bioactive compounds such as collagen peptide [11], ginsenoside [12], and anthocyanins [13] are beneficial for the prevention and treatment of skin photoaging. Epigallocatechin gallate (EGCG), a primary natural polyphenol derived from green tea, is known for various properties, including anti-inflammatory, antioxidant, anti-tumor, and anti-bacterial [14,15]. An increasing amount of research indicates its potential in repairing photoaged skin.

**Figure 1:**
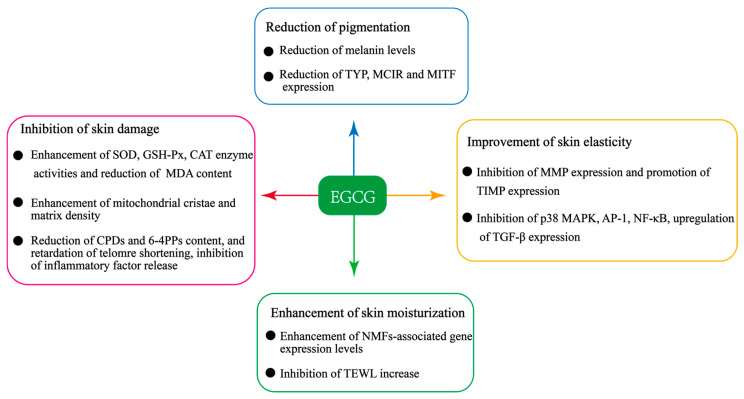
Beneficial effects of EGCG for repairing photoaged skin. TYR, tyrosinase; MC1R, melanocortin 1 receptor; MITF, microphthalmia-associated transcription factor; CPDs, cyclobutane pyrimidine dimers; 6-4PPs, pyrimidine-6,4-pyrimidone photoproducts; CAT, catalase; GSH-Px, glutathione peroxidase; SOD, superoxide dismutase; MDA, malondialdehyde; NMFs, natural moisturizing factors; TEWL, transepidermal water loss.

This review summarizes reports on the beneficial effects of EGCG on photoaged skin, such as improving skin elasticity, enhancing skin moisturization, inhibiting skin damage, and reducing skin hyperpigmentation (Figure 1). It also discusses various application forms of EGCG for repairing photoaged skin due to its poor stability and low bioavailability. These application forms might offer references for developing and applying EGCG products to repair photoaged skin.

## 2. Structural Characterization and Bioavailability of EGCG

**Figure 2:**
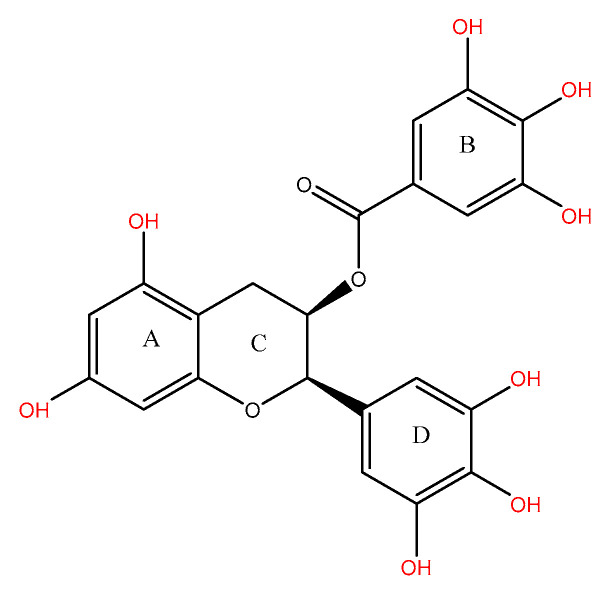
Structure of epigallocatechin gallate (EGCG).

EGCG consists of one molecule each of gallic acid and catechin, featuring a chemical structure that includes a benzenediol ring (A) connected to a tetrahydrophan moiety (C), a pyrogallol ring (D), and a galloyl group linked to the B ring. EGCG contains eight phenolic hydroxyl groups (Figure 2) and exhibits strong anti-inflammatory and antioxidant properties, with the B-ring identified as the primary site for antioxidant activity [10]. This property is further enhanced by the trihydroxy structure present in the D-ring [10,16]. According to the Lipinski rule of 5, EGCG has a logP value of less than 5; however, it possesses eight hydrogen bond donors and has a molecular weight of 458.372 g/mol, which is anticipated to result in poor oral bioavailability [17].

Oral administration is the most commonly used form of EGCG. The plasma EGCG concentration peaks 90 min after oral administration and disappears 12 h later in healthy volunteers [18]. The serum peak concentration reached about 1.4–2.4 h after drinking decaffeinated green tea, with a half-life of 5.0–5.5 h [19]. The metabolokinetic half-life of EGCG in humans appears to be shorter than in rats [20]. After oral administration of EGCG in rats, less than 5% of the dose is absorbed into the bloodstream [21]. The main reasons that limit the utilization of EGCG when taken orally are poor stability and low absorption efficiency of EGCG in vivo. EGCG is very unstable in the internal digestive system, such as the oral cavity, stomach, and intestine; especially under the alkaline condition of the intestine, free EGCG retention is rapidly reduced [22]. At the same time, numerous unfavorable metabolisms of EGCG also occur in the gastrointestinal tract and liver [23]. Catalytic reactions by enzymes such as catechol-O-methyl transferase (COMT), sulfatases, and β-glucuronidases located in the intestine, liver, or target organs can alter the structure of EGCG, resulting in reduced stability [23]. In addition, intestinal microorganisms are also involved in the metabolism and degradation of EGCG [22]. EGCG is completely metabolized in the porcine cecum within 4–8 h [24]. In a clinical study, after the repeated drinking of black tea, the EGCG content in volunteers’ blood, urine, and feces was only 0.14% [25]. This result suggests that EGCG undergoes degradation and adverse metabolism in the digestive system and is, therefore, unstable in the body.

Another important reason for limiting the utilization of EGCG when taken orally is the low absorption efficiency, most of which does not enter the blood but enters the excretory system through bile and is excreted by feces [22]. At the same time, regulated by the efflux pump of multidrug resistance-associated protein (MRP), EGCG re-enters the intestine from the blood through efflux, thereby reducing the absorption of EGCG by body cells [22,26]. Based on the above two main reasons, only a small part of EGCG is absorbed by the body after oral administration, thus limiting its biological function.

In addition to oral administration, topical use is an application form of EGCG. Topical formulations appear to be more effective in delivering EGCG than oral administration [27], as the gastrointestinal environment and in vivo metabolism do not affect the stability of topical formulations. However, topical formulations have also shown low bioavailability, mainly due to the low efficiency of transdermal administration and automatic photodegradation of EGCG [28]. The penetration of EGCG into the skin stratum corneum is a key obstacle due to its high molecular weight [29]. Natural sunlight exposure can cause photodegradation of EGCG in the environment, greatly reducing its content and activity, which limits its clinical application for external use [30]. Additionally, factors such as antioxidants, temperature, and pH can also affect the stability of EGCG in topical preparations [28,30].

## 3. Beneficial Effects of EGCG in Repairing Photoaged Skin

Studies have demonstrated the reparative effects of EGCG on UV-induced photoaged skin. The beneficial effects of EGCG on photoaged skin primarily include improvements in elasticity, enhanced moisturization, inhibition of damage, and reduction in pigmentation. The main molecular targets of EGCG for repairing photoaged skin are outlined in Table 1.

### 3.1. EGCG Improves the Elasticity of Photoaged Skin

UV radiation can accelerate the degradation of skin collagen, leading to a reduction in skin elasticity. UV radiation and the resulting ROS co-promote epidermal growth factor receptor (EGFR) [49], which in turn activates mitogen-activated protein kinases (MAPK) signaling pathways composed of extracellular signal-regulated kinase (ERK), C-Jun N-terminal kinase (JNK), and P38 [4,50]. The activation of JNK and P38 pathways increases the expression of the *C-Jun* gene, while activated ERK enters the nucleus to stimulate the expression of the *C-Fos* gene [50,51]. Consequently, MAPK pathway activation leads to the activation of activator protein 1 (AP-1), composed of C-Jun and C-Fos, directly promoting matrix metalloproteinases (MMPs) and cathepsin synthesis [52]. Additionally, UV light can activate the nuclear transcription factor-κB (NF-κB) signaling pathway and enhance MMP-related gene expression. Normally, NF-κB combines with inhibitive κB (IκB) to confine NF-κB within the cytoplasm [50]. However, in response to UV irradiation, the IκB kinase phosphorylates and degrades IκB with increased ROS levels in vivo, allowing the translocation of NF-κB to the nucleus, leading to increased MMP transcription [50]. Furthermore, tumor necrosis factor-α (TNF-α) also induces MMP expression [53,54]. Finally, the increase in MMPs and cathepsin caused by UV radiation increases the degradation of skin collagen [51], which gradually loosens the skin collagen network layer and decreases skin elasticity.

UV radiation can also hinder the synthesis of skin collagen, leading to a decrease in skin elasticity. Transforming growth factor-β (TGF-β) plays a crucial role in regulating collagen synthesis by binding to specific receptors, such as TGF-βI receptor (TβRI) and TGF-βII receptor (TβRII). The binding of TGF-β binds to TβRII activates the intrinsic serine/threonine kinase activity of TβRI and subsequently phosphorylates the transcription factors Smad2 and Smad3, thereby promoting collagen synthesis [55]. Following exposure to UV light, the expression of TβRII is reduced while the expression of inhibitory factor Smad7 is increased. This results in inhibition of activation of transcription factors Smad 2 and Smad 3, consequently reducing collagen synthesis [56]. UV radiation activates AP-1, which in turn inhibits the TGF-β pathway and reduces collagen synthesis [51]. Furthermore, c-Jun can bind to Smad3 to form a complex that blocks Smad3 from binding to specific cis-acting progenitors and reduces collagen synthesis [57]. Ultimately, decreased collagen synthesis due to UV radiation leads to a reduction in skin elasticity.

**Figure 3:**
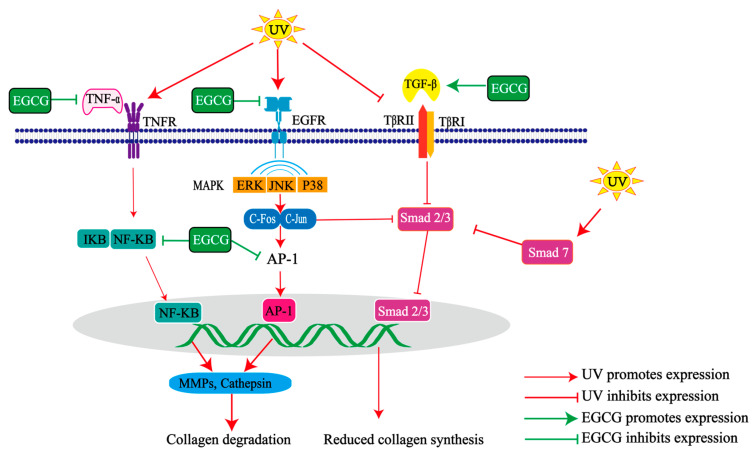
Mechanisms of UV damage and EGCG repair of collagen network layers in photoaged skin. EGCG inhibits activated protein-1 (AP-1) and nuclear transcription factor-κB (NF-κB), contributing to decreased expression of matrix metalloproteinases (MMPs) and increased collagen synthesis through the epidermal growth factor receptor (EGFR), mitogen-activated protein kinase family (MAPK) signaling pathway, tumor necrosis factor-α (TNF-α), and transcriptional growth factor-β (TGF-β). AP-1 also inhibits collagen synthesis by inhibiting the transcription factors Smad 2 and Smad 3. ERK, extracellular signal-regulated kinase; p38, p38 mitogen-activated protein kinase; JNK, C-Jun N-terminal kinase; TβRI/II, transforming growth factor-β type I/II receptor; TNFR, tumor necrosis factor receptor.

EGCG has been found to inhibit the increase in collagen, elastin degradation, and the decrease in collagen synthesis caused by UV radiation, thus preventing the reduction in skin elasticity resulting from UV exposure (Figure 3). EGCG reduces the expression of MMPs in UV-irradiated human skin fibroblast (HSF), human dermal fibroblast (HDF), human keratinocytes (HaCaT) cells, artificial skin, zebrafish, and hairless mouse dorsal skin [32,33,35,39,41,44,46]. Furthermore, treatment with EGCG resulted in an increased expression of TGF-β in UV-irradiated HSF cells, as well as elevated levels of tissue metalloproteinase inhibitors (TIMPs) in both UV-irradiated HSF cells and artificial skin [32,35]. Moreover, EGCG inhibited the expression of p38 MAPK, AP-1, NF-κB, and C-Fos transcription in UV-irradiated zebrafish and HSF cells [39], reducing collagen degradation. At present, it remains unclear whether the inhibitory effect of EGCG on AP-1 is attributed to JNK activation or ERK activation. It has been reported that the inhibition of AP-1 by EGCG is due to its suppression of JNK activation and not related to ERK activation [58]; others have suggested that it is due to its inhibitory effect on the phosphorylation of ERK and mitogen-activated protein extracellular kinase (MEK) and Src [31].

The results of animal studies showed that local application of an EGCG preparation could effectively reduce the epidermal layer thickness of the dorsal skin of hairless mice irradiated by UVB, improve the tightness of skin collagen fibers, reduce the formation of cavities, and increase collagen content [41,43]. Clinical trials have also demonstrated the therapeutic potential of EGCG in addressing skin photoaging caused by UV exposure. A randomized controlled clinical trial involving 50 healthy white adults aged 18–65 revealed that oral green tea extract (GTE) and vitamin C could mitigate the degradation of skin elastin fibers due to UV irradiation, thereby preserving skin elasticity [47]. In GTE, EGCG is present in higher amounts, and it is suggested that EGCG may possess similar therapeutic potential. It is important to note that the exact components within GTE responsible for inhibiting elastic fiber degradation remain unclear, particularly due to the addition of vitamin C and the complex composition of GTE used in the experiment. While previous studies have not found evidence supporting the ability of vitamin C to promote collagen synthesis, it can be inferred that any observed improvement in skin condition among subjects is attributable to the beneficial properties of GTE. However, this does not discount a potential role for vitamin C. These experiments confirm that EGCG may hold promise in increasing collagen and elastin content in the skin following UV irradiation, thus aiding in repairing ECM degradation associated with skin photoaging and ultimately effectively preventing and addressing increased skin sagging and wrinkles.

### 3.2. EGCG Enhances Skin Moisturization

In addition to reducing the elasticity of photoaged skin, UV exposure also reduces skin moisture, resulting in skin dryness and peeling of epidermis. Natural moisturizing factors (NMFs) composed of hyaluronic acid (HA) and filaggrin affect the moisturizing barrier of the skin, with HA being a key regulator of skin moisture levels. Acute UV irradiation can activate *HYAL* gene and increase its expression level, leading to increased HA degradation [37], while chronic UV irradiation can downregulate HA synthesis [59]. Whether promoting HA degradation or inhibiting its synthesis, UV irradiation will eventually reduce the NMF content, destroying the moisturizing barrier and decreasing skin moisture.

Cell experiments have demonstrated that EGCG can reduce *HYAL* gene expression and HA degradation in UVB-irradiated HaCaT cells and increase the expression of NMF-related genes in cells, thereby maintaining the skin water barrier [37]. Animal experiments have shown that dietary supplementation of EGCG inhibits the increase in rat transepidermal water loss (TEWL) caused by UVB irradiation, thereby reducing the skin water loss of rats [60]. Clinical trials have indicated that EGCG improves UV-damaged skin moisture. In a clinical trial involving 45 women aged 40–65 whose back skins were exposed to blue light simulation while orally consuming GTE containing 50% EGCG, they had improved skin elasticity, hydration, and structure compared to the control group. This result suggests that EGCG may enhance women’s skin elasticity, hydration, and structure while alleviating skin photoaging effects [48].

### 3.3. EGCG Inhibits Photoaged Skin Damage

EGCG can repair UV-radiation-induced photoaged skin damage primarily by reducing oxidative stress, DNA damage, and inflammation and inhibiting mitochondrial dysfunction.

#### 3.3.1. EGCG Reduces Oxidative Stress

Under the stimulation of UV radiation, the body undergoes oxidative stress and activates its antioxidant system. During this process, nuclear factor erythroid 2-related factor 2 (Nrf2), an essential transcription factor located in the cytoplasm responsible for activating the antioxidant system, translocates into the nucleus to promote the expression of antioxidant enzymes such as heme oxygenase-1 (HO-1), thereby mitigating oxidative stress [61,62,63]. However, UV radiation induces DNA damage and downregulates Nrf2 expression through transcriptional inhibition, which leads to reduced Nrf2 activation; this makes it challenging to inhibit UV toxicity [62]. Additionally, BTB and CNC homolog 1 (Bach1) compete with Nrf2 by inhibiting the synthesis and expression of antioxidant enzymes within the nucleus [64].

**Figure 4:**
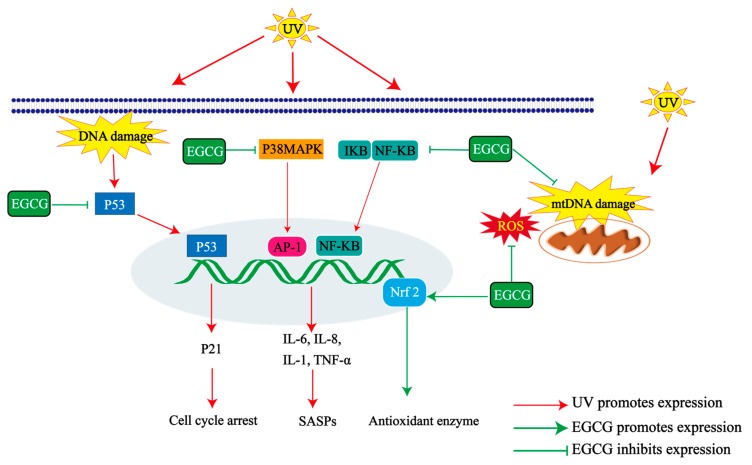
Mechanisms of UV damage and EGCG repair in photoaged skin. EGCG inhibits the release of pro-inflammatory mediators such as tumor necrosis factor α (TNF-α) and interleukin-6/8/1 (IL-6/8/1) through p38 mitogen-activated protein kinase (P38MAPK) and nuclear transcription factor kappa B (NF-κB) and inhibits senescent cells from exhibiting senescence-associated secretory phenotypes (SASPs). EGCG avoids cell cycle arrest by inhibition of DNA damage and expression of P53 and P21. EGCG decelerates mitochondrial dysfunction and maintains the integrity of mitochondrial DNA (mtDNA). EGCG increases the antioxidant enzyme activity and inhibits reactive oxygen species (ROS) content through nuclear factor erythroid 2-related factor 2 (Nrf2).

EGCG is a potent antioxidant that reduces elevated ROS levels caused by UV irradiation, effectively inhibiting decreased activity of antioxidant enzymes and increased production of peroxide products. This ultimately reduces oxidative stress and prevents skin photoaging (Figure 4). Following UV irradiation, EGCG has been shown to increase superoxide dismutase (SOD) and glutathione peroxidase (GSH-Px) activities in HSF and HDF cells while inhibiting malondialdehyde (MDA) content increase [32,34,39]. Furthermore, animal experiments have demonstrated antioxidant properties associated with EGCG. Topical preparations containing EGCG or green tea polyphenols (GTPs) have been found to reduce hydrogen peroxide and MDA levels in skin tissue from hairless mice exposed to UVB radiation while also increasing SOD, catalase (CAT), and GSH-Px enzyme activities [41,42].

Additionally, GTPs promote Nrf2 accumulation within the nucleus while facilitating Bach1 export from the nucleus, subsequently resulting in Bach1 degradation upon entry into the cytoplasm, which slows down its inhibitory effect on Nrf2, leading to increased antioxidase expression [42]. EGCG is a primary component within GTPs, consistent with findings suggesting its potential impact on the Nrf2 pathway [61]. However, the authors did not provide detailed information regarding GTP content or specific components [42].

#### 3.3.2. EGCG Alleviates DNA Damage and Inflammation

Excessive production of ROS due to UV exposure can result in various types of cellular DNA damage, including structural damage, mutations in proto-oncogenes and oncogenes, and even the development of skin cancer. UVB irradiation induces the formation of cyclobutane pyrimidine dimers (CPDs) and pyrimidine-6, 4-pyrimidine photoproducts (6-4PPs), which directly damages DNA and leads to the shortening of telomeres in cell chromosomes [65,66]. On the other hand, UVA indirectly damages DNA through its interaction with cellular chromophores [67].

As photoaged cells proliferate, the number of cells in the G1 phase increases to mitigate the effects of DNA damage caused by UV exposure. This subsequently leads to ECM degradation and ROS production while indirectly stimulating pyruvate signaling and activating a skin inflammatory response [68]. DNA damage and disorders in cell metabolism can lead to increased inflammatory response and expression of inflammatory factors, resulting in inflammatory aging [69]. Inflammatory responses are primarily triggered by the NF-κB and p38MAPK pathways that transmit signals through the neuroendocrine system, ultimately leading to the synthesis and release of a variety of pro-inflammatory mediators such as interleukin, histamine, serotonin, and prostaglandin E2 (PGE2) [68,70]. In particular, PGE2 prevents collagen production and induces the expression of MMPs, thereby affecting the EPK pathway [71]. The release of these mediators increases cellular capillary permeability, leading to infiltration and activation of neutrophils and other phagocytes [72]. At the same time, the increase in inflammatory factors stimulates the overactivation of immune cells, which, together with senescent cells, trigger senescence-associated secretory phenotypes (SASPs), affect the skin microenvironment, and continue to promote cell aging [69].

EGCG reduces DNA damage caused by UV radiation (Figure 4). Pretreatment with a nano-formulated EGCG preparation reduced the increase in CPD and 6-4PP content in HaCaT cells induced by UVB irradiation, thereby slowing down the DNA damage of the cells [36]. Additionally, EGCG can inhibit telomere shortening and cell cycle arrest induced by UVA irradiation [32]. It also reduced the frequency of *HPRT* gene mutation in HSF cells while increasing the senescence and apoptosis of HSF cells induced by UVA irradiation [73]. Morley’s study found that drinking 540 mL of green tea could reduce the DNA damage of peripheral blood cells of 10 volunteers aged 30~57 years after UVA irradiation [74].

In addition, EGCG can counteract the skin’s inflammatory response during photoaging (Figure 4). Studies have demonstrated that tea polyphenols (TPs) emulsified with sodium carboxymethyl cellulose can effectively inhibit acute inflammatory cell infiltration in the skin of hairless mice irradiated by UVB [42]. Furthermore, EGCG or Gcl-EGCG can inhibit the production of inflammatory factors IL-6, IL-8, and IL-1 [45]. Additionally, EGCG decreased the expression of inflammatory factors TNF-α, IL-1α, and IL-6 in zebrafish and HSF cells induced by UVA and UVB radiation [39]. Tea extract with EGCG as its main component inhibited the UV-induced expression of IL-6 and IL-8 [46]. Adding EGCG to a normal diet increased the minimum erythema dose (MED) of the skin of hairless rats after UV irradiation, reduced the severity of skin sunburn caused by UV radiation, and increased the skin’s tolerance to UV radiation [75]. A meta-analysis of recent literature regarding catechins for skin erythema demonstrates that regular intake of green tea catechins (GTCs) reduced skin erythema inflammation caused by UV exposure [76]. In a clinical trial conducted by Rhodes [77] on 16 healthy white volunteers exposed to a UV simulator composed of UVA and UVB radiation sources to induce skin photoaging damage, it was found that oral administration of GTE containing 40.3% EGCG and vitamin C for 12 weeks effectively inhibited UV irradiation-induced skin inflammation and that the level of inflammatory mediator 12-hydroxyeicosatetraenoic acid was reduced while PGE2 did not change. The skin’s inflammatory response is influenced by the concentration of catechins in the skin and the type of metabolite after oral supplementation. Individual differences in skin response to EGCG preparations should be acknowledged as they may affect efficacy.

Topical skin application of GTC was more effective than oral administration, possibly due to avoiding the effects of early digestion. Topical application of an EGCG preparation before acute UVA irradiation reduces the formation of sunburned cells and increases leukocyte infiltration in rats [78]. Furthermore, topical application of an EGCG preparation can inhibit UVB-induced leukocyte infiltration in mice [79].

While previous experiments confirmed the potential benefits of EGCG in inhibiting UV-induced inflammatory responses, a clinical study conducted by Farrar et al. [80] showed contradictory results. The study recruited 50 white adults aged 18–65 years for UV exposure using a solar simulator, with the treatment group taking 1080 mg of GTE containing 40% EGCG and 100 mg of vitamin C daily for three months. It was found that this treatment did not reduce UV-induced skin erythema, leukocyte infiltration, and eicosanoid levels compared to the control group. The results indicated that this treatment was ineffective in altering the skin inflammatory response caused by UV exposure, possibly due to individual variations in response to GTE supplementation and gender differences among study subjects.

#### 3.3.3. EGCG Inhibits Mitochondrial Dysfunction

The skin is a highly metabolic organ that relies on the constant renewal of keratinocytes and fibroblasts to maintain the skin barrier. Mitochondrial respiration serves as the energy source for the continuous renewal of skin cells. Mitochondrial dysfunction leads to the aging of skin cells due to insufficient energy, a key characteristic of skin cell aging. Additionally, maintaining the integrity of mitochondrial structure and function improves the maintenance of skin collagen fiber density [81]. ROS, produced by UV radiation, primarily target mitochondrial DNA (mtDNA), leading to mutations due to the lack of protective histones and mechanisms. This ultimately results in mitochondrial dysfunction [82]. In mouse skin cells, UV radiation causes mitochondrial degradation, reducing mitochondrial ridge and matrix density while increasing vacuolation, further contributing to mitochondrial dysfunction. Carnitine acetyltransferase (CRAT) is essential in acetyl-CoA homeostasis within the mitochondrial matrix. The content of CRAT in photoaged skin is reduced, the mitochondrial ROS level of skin cells is increased, and the skin collagen fiber density is reduced after CRAT knockdown [83]. Under oxidative stress, mitochondrial energy requirements vary quantitatively and qualitatively, mainly related to their regulatory roles in mitochondrial autophagy and dynamics. Abnormal regulation of mitochondrial dynamics by UV radiation leads to excessive division of mitochondria, promoting overproduction of ROS and cell apoptosis. The disruption of mitochondria autophagy results in an imbalance in energy homeostasis and cellular dysfunction, ultimately leading to cell aging [84].

EGCG is a mitochondria-targeting molecule with properties that prevent mitochondrial degradation and induce mitochondrial biogenesis [85]. Thus, it may support continuous skin cell renewal by protecting mitochondrial structure and function and reducing mitochondria-mediated apoptosis (Figure 4). Animal experiments have demonstrated that the local application of EGCG or its primary compound, gallocatechin gallate (GCG), effectively preserves the mitochondrial structural integrity of the back skin cells of hairless mice irradiated by UVB. This preservation increases the density of the mitochondrial ridge and matrix while reducing vacuolation, thereby inhibiting mitochondrial dysfunction in skin cells [43,86]. Furthermore, long-term treatment of H_2_O_2_-induced HDF cells with 12.5 µM EGCG resulted in more complete mtDNA and improved mitochondrial membrane potential [87]. However, studies on mitochondrial function and related enzyme activities in mitochondria are still lacking. Therefore, the mechanisms through which EGCG protects mitochondrial structure and function remain unclear.

EGCG can inhibit the decrease in mitochondrial membrane potential and increase in apoptosis of human lens epithelial cells stimulated by UVB. It can also reduce the expression of Bax (bcl-2 associated X protein), cytochrome c, caspase-3, and caspase-9 in these cells while increasing the expression of Bcl2 (B-cell lymphoma 2). These findings indicate that EGCG can inhibit UVB-induced apoptosis of human lens epithelial cells through a mitochondria-mediated apoptosis signaling pathway [88]. Additionally, EGCG can protect cells through mitochondria-mediated apoptosis by inhibiting the release of cytochrome c and activation of caspases [89]. These mechanisms remain to be further explored in the role of EGCG in repairing UV-induced skin aging.

### 3.4. EGCG Reduces Pigmentation of Photoaged Skin

Melanin is a biological pigment that determines the color of skin, hair, and eyes based on its content as well as its physical and chemical properties [90]. Human epidermal melanocytes (HEMs) produce melanin at the junction between the epidermis and dermis and play a crucial role in transporting melanin to HaCaT cells [1,90]. While melanin protects against radiation damage to the skin, abnormal local skin pigmentation can lead to uneven skin tone, and excessive accumulation of melanin greatly increases melanoma risk [91].

UV radiation serves as an external factor influencing melanin production within the skin, which promotes HEM proliferation, thereby increasing melanin content and its transport within epidermal layers. Additionally, exposure to UV light acts as a potent inducer for expressing proopiomelanocortin (POMC)-derived peptides such as adrenocorticotropic hormone (ACTH) and α-melanocyte stimulating hormone (α-MSH) [92,93]. ACTH and α-MSH bind to the melanocortin 1 receptor (MC1R), activating a signaling pathway dependent on cyclic adenosine monophosphate (cAMP) [94]. This activation stimulates pathways involved in melanin production by upregulating microphthalmia-associated transcription factor (MITF), which then enhances expression levels of three enzymes: tyrosinase (TYP) and tyrosinase-related proteins TRP1 and TRP2, all essential for melanin synthesis. This process facilitates the conversion of tyrosine into additional melanin [95].

EGCG exhibits potent anti-melanin properties and effectively inhibits the activity of TYP enzyme, owing to its strong biological activity [96,97]. It binds to the active center of TYR and interacts with copper ions and key amino acid residues, thereby reducing TYR activity [98]. EGCG can effectively reduce the melanin content and TYR activity without affecting the activity in B16F10 mouse melanoma cells, and it can also inhibit UVA-induced melanosome formation in B16F10 cells [99]. EGCG does not directly affect TYR activity or inactivate α-MSH nor inhibit α-MSH binding to MC1R for melanin synthesis inhibition. Instead, it downregulates the expression of MC1R, MITF, and TYR-related proteins to inhibit melanin formation [91].

In similar experiments, HaCaT and HEM cells were exposed to UVA and TP treatments. The results showed that TPs reduced melanin content, tyrosinase activity, and the expression of MITF, TRP1, and TRP2. Additionally, TPs directly inhibited α-MSH expression [100]. However, the composition of TPs was not described in this experiment, and the main substances in TPs that inhibit melanin synthesis are unknown. Nevertheless, this study confirmed that EGCG, as the main active component of TPs, may have the potential to inhibit melanin production by HaCaT and HEM cells after UV irradiation and may have a preventive effect on local pigmentation caused by skin photoaging.

## 4. Application Forms of EGCG in Repairing Photoaged Skin

Due to the instability and low bioavailability of EGCG, high-dose formulations are often used to increase its bioavailability. ADME experiments in rats and dogs have shown that high doses of EGCG (250 mg/kg body weight and 400 mg/kg body weight) can increase bioavailability from 1.6% to 13.9% compared to low doses [101]. Volunteers who took 800 mg of EGCG daily for four weeks experienced a 60% increase in free plasma EGCG [102]. However, it has been observed that high doses of GET (10–29 mg/kg/day) in mice may lead to hepatitis [103,104], indicating potential harm associated with the use of high doses. Therefore, it is essential to explore the use of EGCG with both high bioavailability and beneficial effects on the body, as well as an appropriate application form meeting these requirements, such as effective delivery systems, modified EGCG, and combination with other substances.

An effective delivery system for EGCG can inhibit its degradation and improve its bioavailability by constructing an EGCG delivery carrier, modifying EGCG itself, or combining it with other substances. Nano-drug delivery carriers have been found to prolong the intestinal retention time of EGCG, enhance its absorption efficiency by skin and intestinal mucosa, and even alter its cellular uptake mechanism. Common nano-delivery systems for EGCG mainly consist of nanoparticles supported by proteins, polysaccharides, and lipids (Table 2). Proteins can bind to EGCG and enhance the efficiency of EGCG absorption; commonly used encapsulated proteins include gelatin, lactoglobulin, casein, etc. [104]. Chitosan, a mostly used polysaccharide carrier, exhibits good biocompatibility and bioactivity encapsulated within chitosan capsules; EGCG becomes more stable with a better-sustained release effect in simulated gastroenteric fluid [105]. Encapsulation within liposomes improves the efficiency of EGCG’s passage through cuticle barriers while reducing water loss from the cuticle [106], ultimately enhancing oral bioavailability [107] and antioxidant activity [108].

Modifying EGCG enhances its bioavailability and bioactivity by altering the number of phenolic hydroxyl groups and the polarity of the acyl portion of EGCG [109]. These modifications typically involve acetylation, glycosylation, and esterification. The antioxidant capacity of EGCG was partially reduced after both esterification [109] and acetylation [110] modifications, but lipid solubility and stability were improved. The antioxidant properties of glycosylated EGCG did not seem to be reduced, and it was also found that glycosylation of EGCG led to specific selection for HaCaT cells [45].

Furthermore, combining EGCG with other substances, such as hyaluronic acid, olive oil, proteins, and antioxidants, improves its bioavailability and bioactivity. Hyaluronic acid possesses potent antioxidant properties and offers effective protection against HaCaT cells. Olive oil enhances mitochondrial function and reduces skin oxidative damage [111]. Proteins serve as carriers for polyphenols in the gastrointestinal tract [112], while collagen, an essential component of skin tissue, exhibits improved stability when combined with EGCG [113]. Due to its susceptibility to oxidation, various antioxidants such as vitamin C, vitamin E, and alpha-lipoic acid are often added to cosmetic creams and used in clinical trials alongside EGCG. In aqueous solution, vitamin C and α-lipoic acid can stabilize the structure of EGCG and inhibit its photodegradation of EGCG to enhance intestinal absorption efficiency [114,115]. Additionally, it has been observed that dietary preparations inhibiting MRP activation may decrease exocytosis, leading to increased absorption of EGCG in the small intestine [116] while also inhibiting COMT structural modifications, thereby enhancing the transport of EGCG [117].

**Table 2:** Application forms of EGCG for repairing photoaged skin.

EGCG Forms	Other Drugs	Treatment	Results	Ref.
EGCG liposomes	Hyaluronic acid	Culturing HaCaT cells, constructing EGCG nano-transfers, applying UV irradiation and drug treatment; measuring the efficiency of EGCG liposomes through rat skin	Increasing skin penetration efficiency and EGCG deposition	[44]
	-	Culturing HDF cells, constructing EGCG microsomes by film hydration method, applying UVA irradiation and drug treatment	Extending drug release, improving skin penetration efficiency and EGCG deposition	[118]
	-	Construction of solid lipid nanoparticles (SLNs) and evaluation of permeability and antioxidant properties	Improving skin penetration efficiency and stabilizing antioxidant properties	[106]
EGCG nanoparticles	Olive oil	Cultured HaCaT cells and hairless mice, constructed EGCG polymer nanoparticles, EGCG lipid nanoparticles, and EGCG emulsion nanoparticles, and applied UVB irradiation and drug treatments	Promoting EGCG skin penetration efficiency and stability	[36]
	-	DMBA-induced DNA damage in mouse skin tissues; construction of EGCG-PLGA nanosomes for topical application to mouse interscapular skin of both shoulder blades	Improving bioavailability and drug delivery efficiency	[119]
EGCG nanoethosomes	-	Sucrose ester-stabilized EGCG nano-ethanol bodies stored for 6 months and then applied to mouse skin; UVB irradiation of mouse skin induces damage	Enhancing skin penetration efficiency and stabilizing EGCG nanosomes	[120]
EGCG	Pentagalloyl glucose	Culturing HDF cells, applying UVA irradiation, and combining two drugs	Increasing extracellular matrix deposition of elastin and collagen	[34]
	Collagen	EGCG-modified collagen-forming couplers, cultured hairless mice, imposed UVB irradiation and drug treatment	Improving antioxidant properties, more comprehensive effect of combined use	[41,113]
	Soybean protein	Polymerization of EGCG with amino acid residues of soybean 7S globulin and verification of anti-UVB properties by mouse experiments	Inhibiting of UVB-induced apoptosis, inflammatory factors, and MAPK signaling pathways	[114]
TP emulsion	-	Hairless mice were cultured, carboxymethyl cellulose emulsified TPs to make an emulsion, and UV irradiation and drug treatments were applied separately	Stabilizing TPs in an aqueous solution and increasing deposition; inhibiting inflammatory cell infiltration skin thickness; increasing oxidative stress levels	[42]
Catechin	Vitamin C	Prepared as capsules for combined use, volunteers take orally	Stabilizing catechins in the gut and improving antioxidant properties	[77,80]
Glc-EGCG	-	Culture of HaCaT cells, HSF cells, melanocytes, endothelial cells with Glc-EGCG; UVA, UVB irradiation damage cells	Improving EGCG solubility, stability, and antioxidant properties	[45]
ZNp-EG	-	EGCG-loaded zein nanoparticles, measurement of relevant properties	Increasing antioxidant and anti-tyrosinase activity and stability	[121]
GTE (42.4% EGCG)	Quercetin and multi-nutrients	Oral administration to rats and determination of pharmacokinetic data	56% increase in bioavailability and enhanced intestinal absorption efficiency	[116]

Abbreviations: Glc-EGCG, glucosyl form of EGCG; ZNp-EG, zein nanoparticles containing EGCG; GTE, green tea extract; TPs, tea polyphenols.

## 5. Discussion and Perspectives

The skin functions as a protective barrier, maintaining homeostasis for itself and other organs through the cutaneous neuro-immuno-endocrine system [3,92]. Upon exposure to UV radiation, the skin synthesizes various small molecules, including vitamin D_3_, POMC, and nitric oxide, which are subsequently released into circulation to exert beneficial effects on the human body [3]. Vitamin D and lumisterol hydroxyderivatives play a crucial role in protecting the body from UV-induced damage by enhancing cellular viability, promoting antioxidant activity, facilitating DNA repair processes, and activating anti-inflammatory mechanisms [122]. Hydroxylumisterols, a photoproduct of pre-vitamin D_3_, mitigate photodamage induced by UVB radiation in HaCaT cells [123]. Phototherapy is employed in the treatment of various skin disorders and utilizes a range of light sources, including UV, that have demonstrated therapeutic benefits [93]. However, it is essential not to overlook the detrimental impacts of UV exposure on the skin, such as an increased risk of cancer, autoimmune disorders, and skin photoaging. A rational approach to defining the effects of UV radiation on human health while exploring its beneficial aspects seems imperative for future research aimed at preventing skin photoaging.

Skin photoaging is the consequence of premature aging of skin cells following exposure to UV radiation. Among these, HDF cells appear to be the most dominant cell involved in this process [124,125], followed by HEMs, the primary type of epidermal senescent cells. Fibroblasts proliferate slowly in vivo and are prone to the SASP phenotype, leading to inflammatory responses and ECM degradation that destroys the surrounding microenvironment [125]. The decline in HDF function in photoaged skin is mainly attributed to the breakdown of the dermal collagen network [126,127]. The degradation of collagen in photoaged skin results in the destruction of the collagen network, weakening its mechanical strength and a failure to provide physical support for HDFs, and the reduction in the quantity of HDFs ultimately leads to a decline in its function [128]. By inhibiting the expression of MMPs and promoting the expression of TIMPs [33], EGCG delays the degradation of collagen and elastin in photoaged skin, thereby maintaining the collagen network layer structure, which provides a conducive environment for HDF proliferation and function maintenance. HEMs produce melanin to protect adjacent human epidermal keratinocytes (HEKs). Aging HEMs induce skin cell aging by inhibiting the proliferation of surrounding cells and destroying the proliferative role of basal keratinocytes [124]. As senescent skin cells proliferate and accumulate, they release more cytokines, matrix-modifying enzymes, and other molecules that disrupt skin structure and function [124].

ROS play a dual role in skin photoaging due to UV radiation and as an activator of various “harmful” mechanisms within cells [125]. ROS accumulation threatens many biomolecules and organelles within cells, triggering inflammatory responses and DNA damage. Lipids are crucial in maintaining the body’s barrier function and are essential for preserving skin moisture [128]. UV radiation exacerbates lipid oxidation in the skin, disrupting lipid metabolism and promoting skin aging. MDA serves as a marker for assessing the degree of lipid oxidation. EGCG has been shown to reduce MDA levels and maintain lipid homeostasis in the skin by enhancing the activity of antioxidant enzymes within cells [129].

EGCG has been extensively researched for its potent antioxidant properties, although reports of its pro-oxidative effects have also been reported. When EGCG is introduced into cell cultures, it triggers the production of hydrogen peroxide and low levels of ROS [130]. ROS play a dual effect in living organisms, acting as both signaling molecules that influence cell signaling and as potentially “toxic” molecules. It is suggested that the pro-oxidation effect of EGCG may be beneficial, serving as a second messenger within the cell and thereby helping to mitigate damage caused by UV radiation. However, further comprehensive research is needed to substantiate this claim. In addition, EGCG elicits a wide range of metabolic responses within the body, with these metabolites appearing to play a role in realizing EGCG’s biological function [130]. Different experimental models, with topical or oral administration of GTP and EGCG, have shown the prevention of UV-induced inflammatory responses, collagen network layer breakdown, and oxidative stress [131]. Continued investigation into the biological activity of EGCG and its metabolites in vivo may help elucidate the molecular mechanisms through which EGCG repairs photoaged skin.

The bioavailability of EGCG is influenced by various factors, especially intestinal absorption. The gut plays a crucial role in the absorption, metabolism, and transformation of EGCG, with intestinal microorganisms playing a key role [132]. Short-term GTE supplementation enhances mice’s gut microbiota and cecum/skin metabolome, effectively mitigating their stress response to UV radiation [133]. At the same time, the chemical stability and membrane permeability of EGCG were less than optimal [134]. Furthermore, the skin’s cuticle barrier effect and lipophilicity pose challenges for applying EGCG topical preparations [135].

Drug delivery systems, structural modifications, and combinations with other substances have been employed to enhance the bioavailability of EGCG to varying degrees. Notably, drug delivery systems encapsulate EGCG to bypass the effects of early digestive processes on its stability while improving skin permeability. This explains why there is interest in EGCG nano-drug delivery systems. Nevertheless, there are still unresolved issues regarding the application of EGCG in treating photoaged skin that require further research. These include understanding how oral EGCG affects intestinal absorption and the roles played by MRP and COMT; investigating how gut microbes influence absorption, metabolism, and transformation of EGCG; and finding ways to improve or upgrade EGCG nano-drug delivery system for enhanced stability of the nanosystem to reduce the sudden release of EGCG [23].

While EGCG holds great promise for preventing and treating photoaged skin, its poor stability and low bioavailability limit its clinical application. Studies have focused on enhancing the stability and bioavailability of EGCG in recent years. For example, studies have been conducted to develop medications combining EGCG with common UV absorbers to protect human skin from UV radiation [136]. In addition, rheological testing may assist in developing future drug delivery vehicles [137]. Nevertheless, thorough research on how combinations involving related substances affect the stability and bioavailability of EGCG has yet to be conducted. Furthermore, the mechanism underlying interactions between drugs and EGCG in preventing and treating photoaged skin remains unclear; likewise, gender differences and individual variations among subjects undergoing treatment with EGCG in clinical studies warrant ongoing exploration [138].

## 6. Conclusions

EGCG is an effective candidate for repairing photoaged skin. It effectively addresses various types of skin damage induced by ultraviolet radiation while preserving the normal structure and function of the skin. EGCG can inhibit the expression of MMPs and enhance the expression of TIMPs, thereby delaying the degradation of collagen and elastin in the skin. EGCG can enhance skin moisturization by increasing the expression of NMF-associated genes and inhibiting the increase in TEWL. EGCG also improves antioxidant enzyme activity, inhibits skin inflammation and DNA damage, protects the structural integrity of skin cell mitochondria, and promotes normal mitochondrial function. Furthermore, EGCG also inhibits tyrosinase activity by downregulating the expression of MC1R, MITF, and TYR, thereby inhibiting melanin formation and ultimately alleviating local skin pigmentation. EGCG’s stability and bioavailability can be enhanced through EGCG delivery systems, EGCG modification, and combination with other substances. Among these methods, nanocarriers of delivery systems show particular promise. Further exploration of the mechanism of EGCG repair of photoaged skin and improvement of its bioavailability by constructing nanocarriers could provide safe and efficient skin repair drugs.

## Figures and Tables

**Table 1:** Molecular targets of EGCG for repairing photoaged skin.

Compounds	Model	Treatments	Effects	Ref.
EGCG	HSF cells	EGCG (1–20 µM) pretreatment for 45 min, TNF-α (20 ng/mL) induction for 15 min	↓ MMP-1, ERK ↓ MEK, Src	[31]
		EGCG was applied to the medium for 24 h, and HSF cells were exposed to UVA radiation	↓ MMPs, MDA ↑ TGF-β, TIMP-1, SOD, GSH-Px, CAT	[32]
		Tea extract (0.5 mg/mL) was applied to the culture medium, and HSF cells were exposed to UVB radiation (0–20 mJ/cm^2^)	↓ MMP-2, MMP-9 ↑ Cell viability	[33]
	HDF cells	10 mg/mL EGCG treated HDF cells exposed to UVA (10 mW/cm^2^)	↑ Collagen deposition, insoluble elastin ↓ MDA	[34]
	Artificial skin	EGCG (0.01 mM) was applied to the medium for 6 h and exposed to UVA (20 J/cm^2^) radiation	↓ MMPs ↑ TIMP-1	[35]
EGCG–chitosan nanoparticles	HaCaT cells	EGCG nanoformulations were pretreated for 24 h and exposed to UVB (40 mJ/cm^2^) radiation for 4 h	↓ CPDs, 6-4PPs	[36]
EGCG	HaCaT cells B16F10 cells	EGCG was used to pretreat HaCaT cells for 30 min, UVB (30 mJ/cm^2^) radiation, B16F10 cells were co-treated with αMSH and EGCG (0–100 µM) for 48 h	↑ NMFs, HYAL	[37]
	HaCaT cells	Exposure to UVB (30/60/90 mJ/cm^2^) radiation followed by application of EGCG (200 µg/mL)	↓ IL-6, TNF-α, P53 ↓ P21, C-Fos	[38]
	Zebrafish HSF cells	0–100 μM EGCG used to treat zebrafish, 50 μM EGCG used to treat HSF cells for 72 h followed by exposure to UVR (UVA (10 J/cm^2^) and UVB (30 mJ/cm^2^))	↓ ROS, p38 MAPK, NF-κB, AP-1 ↑ SOD ↓ IL-1α, IL-6, TNF-α, MMP-1	[39]
	B16F10 cells	EGCG (100 µg/mL) was applied to the medium, and the experimental group was treated with the addition of α-MSH	↓ TYR, α-MSH ↓ MC1R, MITF	[40]
EGCG–collagen	Mice	Hairless back treated topically with EGCG–collagen (0.5 mL, 20 mg mL^−1^) followed by exposure to UVB (80 µW/cm^2^)	↑ SOD, GSH-Px ↑ Epidermal thickness, HYP ↓ MDA, TNF-α, MMP-1	[41]
CMC-Na GTPs	Mice	Hairless mice were exposed to UVB (540 mJ/cm^2^) radiation by applying CMC-Na GTPs externally on the back for 30 min	↓ Inflammatory cell infiltration ↑ Nrf2, GSH-Px, CAT, SOD ↓ Bach1, MDA	[42]
EGCG	Mice	Hairless mice topically coated with EGCG preparation (20 µL/cm^2^) and exposed to UVB (fluence rate 1.7 µmol/m^2^ s) 45 min	↓ Melanosomes ↑ Mitochondrial structure ↑ Collagen, elastic fibers	[43]
EGCG nano-transfersomes	HaCaT cells	Application of EGCG (10 mg/mL) and UVB (60 J/m^2^) radiation	↓ MDA, ROS, MMP-2, MMP-9	[44]
Glc-EGCG	Human skin cell models	Exposure to UVA (3 J/cm^2^) and UVB (50 mJ/cm^2^), application of EGCG (0–50 µM) to skin cells	↓ ROS, IL-6, IL-8, IL-1	[45]
Tea extracts	HaCaT cells HDF cells	The cells were incubated with tea extract (50 µg/mL), exposed to UVA (1 J/cm^2^) and UVB (30 mJ/cm^2^), and incubated for 24 h	↑ Hyaluronic acid, collagen ↓ IL6, IL8, MMP1, MMP9	[46]
GTE	Healthy white	50 healthy white (Fitzpatrick skin type I–II) adults aged 18–65 years were randomized to a combination of GTE 540 mg plus vitamin C 50 mg or to placebo twice daily for 12 weeks, exposure to UVR mimicking sunlight (290–400 nm; 5% UVB, 95% UVA)	↑ Fibulin-5	[47]
GTE	Female adults	60 female volunteers consumed green tea polyphenols providing 1402 mg total catechins/d (50% EGCG) for 12 weeks; individual MED was determined for each participant. Irradiation was applied to dorsal skin, with 1.25 MEDs using a blue-light solar simulator	↓ Erythema ↑ Skin elasticity, roughness, scaling, density, and water homeostasis	[48]

Abbreviations: ↑ denotes increase; ↓ denotes decrease; GTE, green tea extract; Glc-EGCG, glucosyl form of EGCG; CMC-Na GTPs, carboxymethyl cellulose sodium green tea polyphenols; HSF cells, human skin fibroblasts; HDF cells, human dermal fibroblasts; HaCaT cells, human keratinocytes; B16F10 cells, B16F10 mouse melanoma cells; MED, minimal erythemal dose; IL-6/8/1α, interleukin-6/8/1α; TNF-α, tumor necrosis factor-α; TGF-β, transforming growth factor-β; EGFR, epidermal growth factor receptor; ERK, extracellular signal-regulated kinase; MEK, mitogen-activated protein extracellular kinase; p38 MAPK, p38 mitogen-activated protein kinase; NF-κB, nuclear transcription factor kappa B; AP-1, activator protein 1; Nrf2, nuclear factor erythroid 2-related factor-2; Bach1, BTB and CNC homology 1; MMPs, matrix metalloproteinases; MMP-1/2/9, matrix metalloproteinases 1/2/9; TIMP-1, tissue inhibitor of metalloproteinase-1; SOD, superoxide dismutase; GSH-Px, glutathione peroxidase; CAT, catalase; MDA, malondialdehyde; CPDs, cyclobutane pyrimidine dimers; 6-4PPs, pyrimidine-6,4-pyrimidone photoproducts; HYP, hydroxyproline; NMFs, natural moisturizing factors; HYAL, hyaluronidase; TYR, tyrosinase; α-MSH, α-melanocyte-stimulating hormone; MC1R, melanocortin 1 receptor; MITF, microphthalmia-associated transcription factor.

## Data Availability

Not applicable.
